# Nationwide study of patients with head and neck paragangliomas carrying SDHB germline mutations

**DOI:** 10.1002/bjs5.39

**Published:** 2018-02-06

**Authors:** J. A. Rijken, N. D. Niemeijer, C. R. Leemans, K. Eijkelenkamp, A. N. A. van der Horst‐Schrivers, A. van Berkel, H. J. L. M. Timmers, H. P. M. Kunst, P. H. L. T. Bisschop, M. F. van Dooren, F. J. Hes, J. C. Jansen, E. P. M. Corssmit, E. F. Hensen

**Affiliations:** ^1^ Department of Otorhinolaryngology/Head and Neck Surgery VU University Medical Centre Amsterdam The Netherlands; ^2^ Department of Endocrinology and Metabolism, Academic Medical Centre University of Amsterdam Amsterdam The Netherlands; ^3^ Department of Endocrinology and Metabolic Diseases Leiden University Medical Centre Leiden The Netherlands; ^4^ Department of Clinical Genetics Leiden University Medical Centre Leiden The Netherlands; ^5^ Otorhinolaryngology/Head and Neck Surgery Leiden University Medical Centre Leiden The Netherlands; ^6^ Department of Endocrinology, University of Groningen University Medical Centre Groningen Groningen The Netherlands; ^7^ Division of Endocrinology, Department of Internal Medicine Radboud University Medical Centre Nijmegen The Netherlands; ^8^ Department of Otorhinolaryngology/Head and Neck Surgery Radboud University Medical Centre Nijmegen The Netherlands; ^9^ Department of Clinical Genetics, Erasmus MC University Medical Centre Rotterdam Rotterdam The Netherlands

## Abstract

**Background:**

Germline mutations in the succinate dehydrogenase B (SDHB) gene predispose to hereditary paraganglioma (PGL) syndrome type 4. The aim of this study was to evaluate the clinical characteristics and outcome of treatment strategies for patients with head and neck paraganglioma (HNPGL) carrying SDHB germline mutations.

**Methods:**

This was a retrospective evaluation of patients with HNPGL carrying SDHB germline mutations in the Netherlands.

**Results:**

In a Dutch nationwide cohort study of SDHB germline mutation carriers, 54 patients with a total of 62 HNPGLs were identified. Forty‐one of 54 patients (76 per cent) visited the outpatient clinic because of associated complaints. Eight patients (15 per cent) had multiple PGLs. One patient (2 per cent) developed a phaeochromocytoma and three (6 per cent) developed a malignant PGL. Twenty‐seven patients (50 per cent) had an operation for their HNPGL and 15 (28 per cent) received radiotherapy. Three patients with HNPGL (6 per cent) were diagnosed with additional non‐paraganglionic tumours.

**Conclusion:**

If an SDHB germline mutation is identified in a patient with HNPGL, the clinician should be aware of the variable manifestations of the SDHB‐linked tumour syndrome, the risk of catecholamine excess, concurrent phaeochromocytoma, and association with non‐paraganglionic tumours.

## Introduction

Paragangliomas (PGLs) of the head and neck are predominantly benign hypervascular tumours that arise from neural crest cells of the autonomic nervous system. Head and neck paragangliomas (HNPGLs) most frequently originate from the paraganglia in the bifurcation of the carotid artery, the jugular foramen, along the vagus nerve or along the tympanic nerve^1^. Other locations are the nasal cavity, paranasal sinuses, parotid gland, cervical sympathetic chain, pharynx, larynx, trachea, aortic arch, ciliary ganglion and thyroid gland^2^. HNPGLs are associated with extra‐adrenal PGLs arising in the thorax and abdomen, predominantly along the sympathetic trunk, and with phaeochromocytomas of the adrenal gland.

These extra‐adrenal PGLs and phaeochromocytomas usually present with signs and symptoms of catecholamine excess^3^. Generally HNPGLs are parasympathetic in origin, and symptoms depend on the localization, tumour size, compression of surrounding structures and associated cranial nerve deficits. Between 4 and 30 per cent of HNPGLs secrete catecholamines^4^ ^5^. HNPGLs can occur spontaneously or as part of a hereditary syndrome. A rapidly expanding number of genes are associated with hereditary PGL. Hereditary PGL syndrome is caused most frequently by genes encoding succinate dehydrogenase (SDH) subunits or co‐factors (*SDHA/B/C/D/AF2* genes). Other associated genes are *RET*, *NF1*, *VHL*, *HIF2A*, *FH*, *TMEM127* and *MAX* 6 7. In the Netherlands, mutations in *SDHD, SDHB* and *SDHAF2* are responsible for most hereditary cases. *SDHD*‐related PGLs are usually characterized by multiple PGLs located predominantly in the head and neck region, with a low frequency of malignancy. In contrast, *SDHB* mutation carriers are reported to develop single PGLs and metastatic PGLs more frequently^8^, ^9^, ^10^, ^11^, ^12^. Recently it has become clear that the *SDHB*‐linked tumour syndrome not only comprises PGLs and phaeochromocytomas, but also non‐paraganglionic tumours such as renal clear cell carcinoma, gastrointestinal stromal tumours (GISTs) and pituitary tumours^6^, ^7^, ^8^, ^9^, ^10^, ^11^, ^12^.

In a recently published nationwide evaluation of 194 *SDHB* mutation carriers^13^, 54 patients (27.8 per cent) were identified with *SDHB*‐linked HNPGLs. In the present study, the clinical characteristics and clinical course, treatment modalities and outcome of these patients with HNPGL linked to *SDHB* mutations were evaluated.

## Methods

Patients with HNPGL were identified in a Dutch nationwide cohort of *SDHB* germline mutation carriers. The genotype and phenotype of this nationwide cohort have been described elsewhere^13^. *SDHB* mutation carriers and patients with PGL were investigated in multiple centres according to structured protocols used for standard care of PGL in the Netherlands^14^ ^15^. Carriers were offered annual clinical surveillance for concurrent HNPGL, concurrent phaeochromocytomas and extra‐adrenal PGLs in departments of otorhinolaryngology and endocrinology. For *SDHB* mutation carriers over 18 years of age, surveillance consisted of MRI of the head and neck region once every 3 years, and MRI or CT of the thorax, abdomen and pelvis once every 2–3 years. At the time of this study there were no national structured protocols for surveillance of *SDHB* mutation carriers aged less than 18 years. Therefore, the method and interval of surveillance in this age category varied between centres.

When HNPGL was diagnosed, treatment or intensified periodic examination was offered, guided by tumour characteristics such as location, size (defined as the largest diameter of the HNPGL on imaging), growth rate, associated symptoms, and patient characteristics such as age, general condition and co‐morbidity, according to local protocols. A wait and scan policy, radiotherapy, surgical resection, or combinations thereof, were possible treatment strategies. Annual biochemical screening included the measurement of adrenaline (epinephrine), noradrenaline (norepinephrine), vanillylmandelic acid (VMA), dopamine (D), metanephrine, normetanephrine and/or 3‐methoxytyramine (3‐MT) in two 24‐h urinary samples, and/or plasma free (nor)metanephrine and/or 3‐MT. In case of excessive catecholamine secretion (any value above the upper reference limit), radiological assessment by MRI or CT of the thorax, abdomen and pelvis and/or [^123^I]metaiodobenzylguanidine (MIBG)scan/PET with 2‐deoxy‐2‐[fluorine‐18]fluoro‐d‐glucose (^18^F‐FDG PET)/^18^F‐l‐dihydroxyphenylalanine (^18^F‐DOPA) PET was performed to identify potential sources of excessive catecholamine production outside the head and neck region. As no histological features of the primary tumour reliably distinguish benign from malignant (HN)PGLs, malignant disease was defined as the presence of metastases (paraganglionic cells in non‐neuroendocrine tissue distant from the primary tumour).

After obtained informed consent, clinical, radiological and genetic data of patients with HNPGL were collected. Duration of the follow‐up was defined as the time from the date of first presentation to the most recent outpatient visit within the study interval.

The study was approved by the medical ethics committee of Leiden University Medical Centre (number P13.161); participating centres complied with their local medical ethics committee requirements.

SPSS^®^ version 20.0 (IBM, Armonk, New York, USA) was used for data analysis.

## Results

### Clinical status

In all, 54 patients, 28 female (52 per cent) and 26 male (48 per cent), with a total of 62 HNPGLs were identified in a nationwide evaluation of *SDHB* mutation carriers^13^. The mean age of diagnosis was 45·9 (range 11–77) years. Sixteen patients (30 per cent) had a positive family history, and 38 (70 per cent) presented with a negative family history (*Table* 1). The mean duration of follow‐up was 7·8 (median 4·5, range 0·1–36·9) years.

**Table 1:** Clinical characteristics of patients with SDHB‐linked head and neck paragangliomas

	Negative family history (*n* = 38)	Positive family history (*n* = 16)
Age at diagnosis (years)*	47 (12–77)	44 (28–83)
Sex ratio (M : F)	19 : 19	7 : 9
Malignant paraganglioma	3	0
Multiple head and neck paragangliomas	6	1
Phaeochromocytoma	1	0
Extra‐adrenal paraganglioma	0	0
Carotid body tumour	11	11
Jugular body tumour	13	1
Vagal body tumour	8	4
Tympanic body tumour	9	1

* Values are mean (range).

### Genetics

In all, 21 different *SDHB* germline mutations were identified (*Table* 2). The most prevalent *SDHB* germline mutations are known as Dutch founder mutations – a deletion of exon 3 (18 patients, 33 per cent) and the c.423+1G>A mutation (11 patients, 20 per cent).

**Table 2:** Details of SDHB germline mutations in patients with a head and neck paraganglioma

cDNA mutation	Protein alteration	No. of patients
Exon 3 deletion†	p.?	18
c.423+1G>A†	p.?	11
c.653G>C	p.(Trp218Ser)	3
c.137G>A	p.(Arg46Gln)	3
c.200+1G>A	p.?	2
c.328A>C	p.(Thr110Pro)	2
c.686_725del	p.(Glu229fs)	1
c.725G>A	p.(Arg242His)	1
c.761C>T	p.(pro254Leu)	1
Exon 1 deletion	p.?	1
Promoter to exon 8 deletion	p. 0	1
Promoter and exon 1 deletion	p.?	1
c.119A>C	p.(Lys40Thr)	1
c.649C>T	p.(Arg217Cys)	1
c.1A>G	p.?	1
c.590C>G	p.(Pro197Arg)	1
c.292T>C	p.(Cys98Arg)	1
c.654G>A	p.(Trp218†)	1
c.380T>C	p.(Ile127Thr)	1
c.418G>T	p.(Val140Phe)	1
c.574T>C	p.(Cys192Arg)	1

† Dutch founder mutations.

### Presenting symptoms

**Figure 1:**
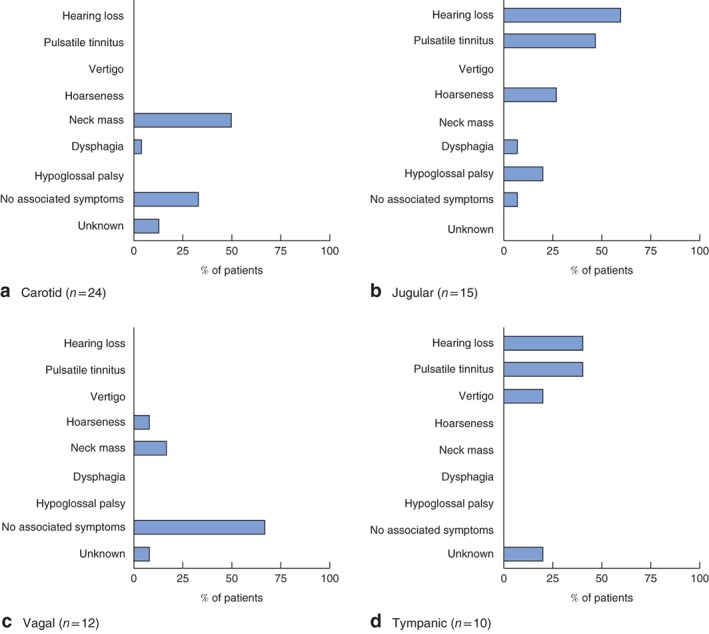
Presenting symptoms in patients with SDHB‐linked head and neck paraganglioma (HNPGL). The proportion of patients with HNPGL who presented with a specific symptom is shown per tumour site. Some patients did not have any symptoms, and the HNPGL was identified coincidentally (as an incidentaloma) or through presymptomatic screening. The single paraganglioma that was diagnosed in the tonsil is not shown. **a** Carotid, **b** jugular, **c** vagal, **d** tympanic

Thirteen patients (24 per cent) had no associated signs or symptoms at the time of diagnosis, and the tumour was identified as a result of presymptomatic screening of known *SDHB* mutation carriers (11 patients) or as an incidentaloma (2). Forty‐one patients (76 per cent) with HNPGL came to medical attention as a result of HNPGL‐associated signs or symptoms. The occurrence and type of presenting symptoms depended on the location of the tumour in the head and neck region (*Fig*. 1).

The majority of patients with tympanic and jugulotympanic PGLs presented with symptoms or signs (20 of 24, 83 per cent), mostly hearing loss and pulsatile tinnitus, whereas the majority with a vagal body PGL (8 of 12, 67 per cent) had no symptoms at the time of diagnosis. Cranial nerve deficit (causing hoarseness, dysphagia and hypoglossal palsy) was most commonly seen in jugular PGLs. Seven carotid body tumours were asymptomatic and the tumour was identified coincidentally (as an incidentaloma) or through presymptomatic testing (7 of 24, 29 per cent). Nineteen *SDHB* carriers with an HNPGL presented with hypertension (19 of 54, 35 per cent).

### Multicentricity and non‐paraganglionic tumours

Multiple PGLs were present in eight (15 per cent) of the 54 patients with HNPGL to a maximum of three concurrent tumours (*Table* 3). In five patients, multiple HNPGLs were discovered during initial imaging. Two patients were initially diagnosed with a solitary HNPGL and developed a second, metachronous, HNPGL during follow‐up. One patient (2 per cent) underwent an adrenalectomy because of a phaeochromocytoma 36 years before the diagnosis of a vagal body PGL. No concurrent extra‐adrenal PGLs were identified in this *SDHB*‐linked HNPGL patient cohort. Three patients (6 per cent) were diagnosed with non‐paraganglionic tumours additional to their HNPGL: a melanoma, a pituitary microprolactinoma and low‐grade B‐cell non‐Hodgkin lymphoma. Although multiple non‐paraganglionic tumours have been shown to be part of the *SDHB*‐linked tumour spectrum, *SDHB* immunostaining was not performed on the non‐paraganglionic tumours found in this study, and so no definitive causal relation with the *SDHB* germline mutation could be established^16^.

**Table 3:** Clinical characteristics, treatment strategies and outcome of patients with multiple SDHB‐linked head and neck paragangliomas

					Treatment				
Sex	*SDHB* mutation	Presenting symptoms	Tumour and location	Age at diagnosis (years)	Strategy	Tumour treated	Age (years)	Outcome	Follow‐up (years)
F	c.653G>C	Unknown	VBTL, PHEO	19	Surgery	PHEO	19	AWD	37
F	Exon 3 deletion	Hearing loss, pulsatile tinnitus, swelling neck, hoarseness	CBTR, JBTR, JBTL	30	RT	JBTL	30	AWD	2
M	c.761C>T	Swelling neck	CBTL, JBTR	33	Surgery	CBTL	33	AWD	28
M	c.423+1G>A	None (PST)	VBTR, JBTL	49	RT	JBTL	56	AWD	6
F	Exon 3 deletion	Pulsatile tinnitus	VBTR, JBTL	49	Surgery	JBTL	49	AWD	15
						VBTR	50		
					RT	JBTL	65		
M	Exon 3 deletion	Pulsatile tinnitus	VBTL, TBTR	52	RT	TBTR	52	AWD	2
F	c.649C>T	Swelling neck	CBTR, CBTL	55	Surgery	CBTL	55	AWD	14
M	c.590C>G	None (incidentaloma)	CBTR, CBTL	56	Watchful waiting			AWD	10

VBTL, vagal body tumour left; PHEO, phaeochromocytoma; AWD, alive with disease; CBTR, carotid body tumour right; JBTR, jugular body tumour right; JBTL, jugular body tumour left; RT, radiotherapy; CBTL, carotid body tumour left; PST, presymptomatic screening; VBTR, vagal body tumour right; TBTR, tympanic body tumour right.

### Location and size

The most frequently found paraganglioma locations within the head and neck region were the jugular foramen (25 tumours: 14 left, 11 right), the carotid bifurcation (24 tumours: 13 left, 11 right) and along the vagal nerve (12 tumours: 6 left, 6 right) (*Table* 1). One patient had a PGL in the right tonsil. Of 24 patients with a jugulotympanic tumour, ten had an isolated tympanic tumour (Fisch type A or B^17^). One of the ten patients with a tympanic PGL had a concurrent carotid body HNPGL.

Mean tumour size at first presentation differed depending on the location of the tumour; the mean size on initial imaging of vagal PGL was 35 (range 4–70) mm, followed by carotid body PGL (28 (4–58) mm), jugular PGL (26 (17–44) mm) and tympanic PGL (10 (4–22) mm).

### Malignancy

Three patients with HNPGL (6 per cent) developed metastatic disease (*Tables* 1 and 4). Initially, these three patients had solitary, seemingly benign, HNPGLs. They developed metastases during follow‐up at 2·2, 9·2 and 31·3 years after initial diagnosis. No clear associations between the occurrence of metastatic disease and genetic factors such as *SDHB* mutation type, or clinical factors such as age of the patient, size of the initial tumour or location of the initial tumour, were found (*Table* 4).

**Table 4:** Clinical characteristics, treatment strategies and outcome of patients with malignant SDHB‐linked head and neck paragangliomas

Sex	*SDHB* mutation	Age (years)*	Age (years)†	Location	Size of primary tumour at initial diagnosis (mm)	Location of metastases	Catecholamine excess at diagnosis	Treatment of primary tumour	Treatment of metastases	Outcome
F	c.418G>T	18	20	Right tonsil	20	Lymph nodes, bone (vertebra)	Urinary level raised (3‐MT); plasma normal	Surgery	Surgery and RT	AWD at age 22 years; subsequently lost to follow‐up
M	c.423+1G>A	48	57	JBTL	Unknown	Bone (vertebra)	Urinary level raised (VMA, D, A, NA); plasma not measured	Surgery and RT (at age 57 years)	None	Died from disease at age 57 years
F	Exon 3 deletion	35	66	CBTL	48	Lymph nodes, bone	Urine negative; plasma not measured	Surgery and RT (at age 66 years) (recurrent CBTL)	None	AWD at age 66 years

* Age at diagnosis of head and neck paraganglioma;

† age at diagnosis of metastatic disease. 3‐MT, 3‐methoxytyramine; RT, radiotherapy; AWD, alive with disease; JBTL, jugular body tumour left; VMA, vanillylmandelic acid; D, dopamine; A, adrenaline (epinephrine); NA, noradrenaline (norepinephrine); CBTL, carotid body tumour left.

### Catecholamine excess

Screening for catecholamine excess was performed at the time of diagnosis and at annual intervals during follow‐up by urine and/or plasma analysis in 52 of the 54 patients. In all, 27 (52 per cent) of these 52 patients tested positive for catecholamine excess during follow‐up. At the time of diagnosis, 14 patients tested positive for adrenaline (epinephrine), noradrenaline (norepinephrine) or their metabolites, and seven tested positive for dopamine or its metabolite. The results of catecholamine screening in the three patients with metastatic HNPGL is outlined in *Table* 4.

### Treatment strategy and outcome

Twenty‐seven patients (50 per cent) had an operation and 15 (28 per cent) received radiotherapy, either as single modality or as adjuvant therapy. In 19 patients (35 per cent) no intervention was performed. Treatment strategies and outcome for patients with a solitary HNPGL are outlined in *Table* 5. Nine of 11 patients with a solitary carotid body tumour showed no evidence of disease after surgery; the other two patients were lost to follow‐up. Of five patients with a solitary jugular body tumour who underwent surgery, four received adjuvant radiotherapy although tumour‐free margins were never achieved at resection. Only two of eight patients with a vagal body PGL received a form of treatment (1 radiotherapy and 1 surgery), and seven of these patients were alive with disease at the end of follow‐up.

**Table 5:** Overall outcome and treatment strategy in patients with a solitary SDHB‐linked head and neck paraganglioma

Tumour location	Overall outcome	Mean follow‐up (years)	Treatment		Outcome			
			Strategy	*n*	NED	AWD	DFD	LTF
Carotid body tumour (*n* = 18)	NED 9	7·8	Watchful waiting	6	–	6	–	–
	AWD 7		Surgery	11	9	–	–	2
	DFD 0		RT	–	–	–	–	–
	LTF 2		Surgery + adjuvant RT	1	–	1	–	–
Jugular body tumour (*n* = 10)	NED 0	7·6	Watchful waiting	4	–	3	–	1
	AWD 8		Surgery	1	–	1	–	–
	DFD 1		RT	1	–	1	–	–
	LTF 1		Surgery + adjuvant RT	4	–	3	1	–
Tympanic body tumour (*n* = 9)	NED 6	8·2	Watchful waiting	2	–	2	–	–
	AWD 3		Surgery	4	4	–	–	–
	DFD 0		RT	2	–	2	–	–
	LTF 0		Surgery + adjuvant RT	1	1	–	–	–
Vagal body tumour (*n* = 8)	NED 0	5·9	Watchful waiting	6	–	5	–	1
	AWD 7		Surgery	1	–	1	–	–
	DFD 0		RT	1	–	1	–	–
	LTF 1		Surgery + adjuvant RT	–	–	–	–	–

NED, no evidence of disease; AWD, alive with disease; DFD, died from disease; LTF, lost to follow‐up; RT, radiotherapy.

## Discussion

This study describes patients with HNPGL identified from a nationwide cohort of *SDHB* mutation carriers. The mean age at diagnosis of an HNPGL in this cohort (45·9 years) was higher than that reported previously, of between 30 and 37 years^8^ ^10^, ^18^. In the Netherlands, tumour screening in *SDHB*‐linked families is advised from the age of 18 years onwards. A later start for tumour screening has been proposed based on statistical models of the age‐dependent penetrance of *SDHB* mutations and, although the mean age in this cohort was relatively high, the youngest patient developed an HNPGL at age 11 years, and an 18‐year‐old patient had already developed PGL metastases. The optimal age to start screening for PGLs in *SDHB* mutation carriers thus remains a subject of debate^19^, ^20^, ^21^.

The majority of patients in this cohort carried a Dutch *SDHB* founder mutation, either a deletion of exon 3 (18 of 54 patients) or the c.423+1G>A mutation (11 patients). Interestingly, the majority of patients with an *SDHB*‐linked HNPGL reported a negative family history (70 per cent), probably reflecting the low penetrance of *SDHB*‐linked PGL syndrome^22^ ^23^. In addition, patients and their physicians may have been unaware that phaeochromocytomas and some non‐paraganglionic tumours such as GISTs, pituitary tumours and renal clear cell carcinomas are part of the tumour spectrum caused by *SDHB* germline mutations^13^.

Patients with *SDHB*‐linked HNPGL had a low risk (8 of 54, 15 per cent) of developing multiple PGLs, in contrast to the risk for *SDHD* mutation carriers (60–79 per cent)^9^ ^10^, ^24^. Only a single patient in this *SDHB*‐linked HNPGL cohort developed a phaeochromocytoma. Thirty‐five years after an adrenalectomy for this tumour, this patient developed a vagal body tumour. No patient with an HNPGL developed extra‐adrenal PGLs, even though these tumours are reported to be relatively prevalent in *SDHB* mutation carriers^12^.

The risk of malignancy in this cohort was also lower than expected, with only three patients (6 per cent) developing metastases. All three presented with an apparently benign solitary HNPGL (located in the tonsil, jugular body and carotid body). Metastatic disease developed during follow‐up, at varying time intervals from initial HNPGL diagnosis (range 2·2–31·3 years). No clear clinical or genetic indicators of malignancy were identified.

Most patients with a carotid, jugular or tympanic body HNPGL had one or more complaints associated with the tumour (*Fig*. 1). Of the 13 patients (24 per cent) without symptoms, vagal body tumours dominated (over 50 per cent). The benefit of detecting asymptomatic, slow‐growing benign PGLs through presymptomatic screening of *SDHB* mutation carriers is uncertain, as intervention by either surgery or radiotherapy may cause more morbidity than the tumour itself. Conversely, early diagnosis seems favourable in growing tumours, catecholamine‐producing tumours and malignant tumours, allowing for timely therapeutic intervention. As the occurrence or type of symptoms does not reliably predict tumour growth, catecholamine excess or malignancy, adequate surveillance of *SDHB* germline mutation carriers is mandatory and should include screening for catecholamines or their metabolites, along with periodic radiological investigation of the abdomen, the pelvic region, thorax, and head and neck region. In patients with *SDHB*‐linked HNPGLs, these regions should be evaluated not only for the occurrence of concurrent PGLs and phaeochromocytomas, but also for *SDHB*‐associated non‐paraganglionic tumours and PGL metastases.

The choice of an optimal treatment strategy for HNPGLs is complex and depends on diverse factors such as the causal gene mutation, patient characteristics (age, condition and preferences) and HNPGL characteristics (localization, size and growth rate, catecholamine excess and associated cranial nerve deficits). Opinions regarding adequate management of HNPGLs have changed over time and vary from centre to centre. Symptoms and risks conferred by the tumour should be weighed against the morbidity of the treatment. As the risk to *SDHB*‐linked patients is not confined to one specific anatomical region or tumour type, these decisions are probably made most appropriately by a dedicated multidisciplinary team.

Genetic counselling and DNA testing is recommended for all patients with HNPGL, as different PGL‐associated genes confer different clinical risks and may warrant different management strategies. If an *SDHB* germline mutation is identified in a patient with HNPGL, the clinician should be aware of the variable manifestations of the *SDHB*‐linked tumour syndrome and, irrespective of the chosen management strategy, periodic surveillance should be performed including screening for catecholamine excess, concurrent PGL or phaeochromocytoma, metastatic PGL and *SDHB*‐associated non‐paraganglionic tumours.

## Disclosure

The authors declare no conflict of interest.
